# Investigating the Association of ApoE Genotypes with Blood-Brain Barrier Dysfunction Measured by Cerebrospinal Fluid-Serum Albumin Ratio in a Cohort of Patients with Different Types of Dementia

**DOI:** 10.1371/journal.pone.0084405

**Published:** 2013-12-27

**Authors:** André Karch, Henrike Manthey, Claudia Ponto, Peter Hermann, Uta Heinemann, Christian Schmidt, Inga Zerr

**Affiliations:** 1 Department of Neurology, Clinical Dementia Centre, University Hospital Göttingen, Göttingen, Germany; 2 Department of Epidemiology, Helmholtz Centre for Infection Research, Braunschweig, Germany; Washington University, United States of America

## Abstract

**Background:**

Since more than a decade ApoE is known to be a strong risk factor for Alzheimer's disease (AD); however, molecular pathways mediating this risk are still unclear. In recent years it has been hypothesized that ApoE might play a role in the disintegration of blood-brain barrier (BBB). In the present study we addressed the question if ApoE genotypes might be associated with BBB function measured by albumin ratio (Q_Alb_) in a large cohort of patients with different types of dementia.

**Methods:**

Five hundred twenty (520) patients with Creutzfeldt-Jakob disease (CJD, n = 350), Alzheimer's disease (n = 71) and cerebral small vessel disease (n = 99) were assessed for their ApoE genotype. BBB function was measured in all patients using Q_Alb_ and was compared between ApoE genotypes. Dominant and additive genetic models were assumed in order to investigate the potential effect of ApoE on BBB function.

**Results:**

We observed no systematic differences in Q_Alb_ between ApoE genotypes within the present study. Increased Q_Alb_ levels were shown for those without E3 allele in the subgroup of CJD patients when assuming a dominant genetic model (p = 0.035). This could not be confirmed for patients with other forms of dementia (p = 0.234).

**Discussion:**

Although there was some evidence for a protective effect of E3 alleles in CJD patients, this study does not support the hypothesis of a systematic role of ApoE genotypes in BBB function in individuals with a diagnosis of dementia. Thus, changes in BBB function do not seem to contribute to the increased risk of cognitive decline associated with certain ApoE genotypes. The interpretation of the results of this study must take into account that BBB function was only assessed by measuring Q_Alb_ which has been shown to be a good marker for overall BBB integrity but might not reflect all qualities of the barrier.

## Background

Presence of one or two E4 alleles of Apolipoprotein E gene (ApoE) is widely known to be a strong risk factor for Alzheimer's disease (AD), but is also associated with other types of cognitive decline [1]–[3]. However, the molecular pathways of these association are still unclear [4]. A recently published experimental study proposed a role of ApoE in the development of BBB dysfunction via cyclophilin A [5]. It was hypothesized that either lack of ApoE3 or presence of ApoE4 alleles might lead to blood-brain barrier (BBB) dysfunction. This is consistent with recent findings demonstrating an association of cerebrovascular dysfunction, BBB dysfunction and AD pathology [6]–[8]. Preliminary analyses of a cohort of patients with cerebral small vessel disease revealed that BBB dysfunction measured by albumin ratio (Q_Alb_) was associated with cognitive decline [9]. To date, there is only a very limited number of small-sized studies available investigating a potential association of BBB function and ApoE genotypes [10]–[13]. Since there is clear evidence for the role of ApoE in the pathogenesis of AD, it cannot be excluded that BBB dysfunction mediated by ApoE plays a role in the development of cognitive dysfunction in AD and other forms of dementia. The aim of the present study was to investigate if ApoE genotypes are associated with BBB function (estimated by cerebrospinal fluid-serum albumin ratio) in a large cohort of patients with different types of dementia.

## Methods

This is a cross-sectional study within the Clinical Dementia Centre Göttingen. All patients initially referred to the centre between 2001 and 2012 and tested for ApoE genotype were considered for inclusion in this study. Individuals with obvious reasons for BBB dysfunction (e.g. acute meningitis) were excluded. All individuals included in this study were diagnosed as patients with AD, Creutzfeldt-Jakob disease (CJD) or cerebral small vessel disease (VD) according to established criteria [14]–[16].

The present study was conducted according to the revised Declaration of Helsinki. Written informed consent was given by all patients (or their legal nexts of kin). Capacity to consent was assessed using a broad range of neuropsychological tests; legal nexts of kin consented on the behalf of participants whose capacity to consent was compromised. Ethics approval for the study as well as for the consent procedure was obtained from the local Ethics committee of the University Hospital Göttingen (11/11/93 with amendments 34/9/07 and 9/6/08).

Genotyping for ApoE alleles was performed by real-time polymerase chain reaction as described previously [17]. BBB function as the outcome of interest was estimated by Q_Alb_ using protein measurement of CSF and serum [13]. Q_Alb_ was defined as the ratio of the albumin concentration in CSF divided by the albumin concentration in blood serum (Q_Alb_ = Alb (CSF in mg/l)/Alb (serum in g/l) [13]. Q_Alb_ was calculated using a diagnostic lumbar puncture at the initial contact with the patient so that timing of Q_Alb_ can be estimated as time of diagnosis.

Data analysis was performed in three steps. First, baseline characteristics were compared between dementia groups using chi square tests, Kruskal-Wallis tests and univariate ANOVAs with respective Post-hoc tests as appropriate. Q_Alb_ was log-transformed for all analyses due to a skewed distribution. In a second step, Q_Alb_ was compared between ApoE genotypes in a univariable analysis using one-way ANOVA. Potential confounders were included in a multivariable analysis using ANCOVA. In case of global differences between ApoE genotypes, Tukey-Post-hoc tests were performed. Predefined subgroup analyses were conducted for the different types of dementia.

Since the underlying genetic model for ApoE has not yet been discovered, we established dominant and additive genetic models to investigate the effect of ApoE in more detail. We used any of the three ApoE alleles as a potential risk allele in both model types. Log-transformed Q_Alb_ values were compared using univariable t-tests and multivariable ANCOVAs (for dominant genetic models) and univariable and multivariable ANOVAs and ANCOVAs (for additive genetic models) [18]. Since these secondary analyses were conducted on an exploratory base only, p values were not adjusted for multiple comparisons. All Q_Alb_ values (means and confidence intervals) presented in this manuscript represent log-transformed values which were back-transformed after analyses.

## Results

A total of 520 demented patients with a diagnosis of CJD (n = 350), AD (n = 71) or VD (n = 99) were included in the present study. ApoE distribution in the study population was similar to the distribution of previous studies performed in the respective disease groups (Table 1) [19]–[21]. ApoE genotypes with one or two E4 alleles were seen more frequently in AD patients than in VD or CJD patients. Consistent with previous knowledge, CJD patients were in average younger at disease onset than AD and VD patients in this study (Table 1). There was no difference in Q_Alb_ between different types of dementia (p = 0.349). However, Q_Alb_ was considerably higher in men than in women (p<0.001).

**Table 1:** Baseline characteristics of study participants by type of dementia.

	All (n = 520)	CJD (n = 350)	AD (n = 71)	VD (n = 99)	p value
**Female sex (n = (%))**	284 (54.6%)	199 (56.9%)	41 (57.7%)	44 (44.4%)	0.078*
**Age (mean (SD))**	66.34 (10.70)	63.91 (10.55)	68.37 (9.81)	69.97 (10.87)	<0.001**
**Q_Alb_ (mean, 95% CI)** ****	6.20 (5.98–6.43)	6.03 (5.76–6.32)	6.29 (5.67–6.97)	6.76 (6.28–7.28)	0.515**
**ApoE genotype**					
*E2/E2 (n = (%))*	5 (1.0%)	4 (1.1%)	0 (0.0%)	1 (1.0%)	
*E2/E3 (n = (%))*	59 (11.3%)	44 (12.6%)	2 (2.8%)	13 (13.1%)	
*E2/E4 (n = (%))*	13 (2.5%)	8 (2.3%)	4 (5.6%)	1 (1.0%)	<0.001***
*E3/E3 (n = (%))*	284 (54.6%)	214 (61.1%)	23 (32.4%)	47 (47.5%)	
*E3/E4 (n = (%))*	133 (25.6%)	72 (20.6%)	32 (45.1%)	29 (29.3%)	
*E4/E4 (n = (%))*	26 (5.0%)	8 (2.3%)	10 (14.1%)	8 (8.1%)	

*Chi square test.

**ANOVA.

***Fisher's exact test.

****Back-transformed means and 95% confidence intervals of the natural logarithm of Q_Alb_.

**Figure 1:**
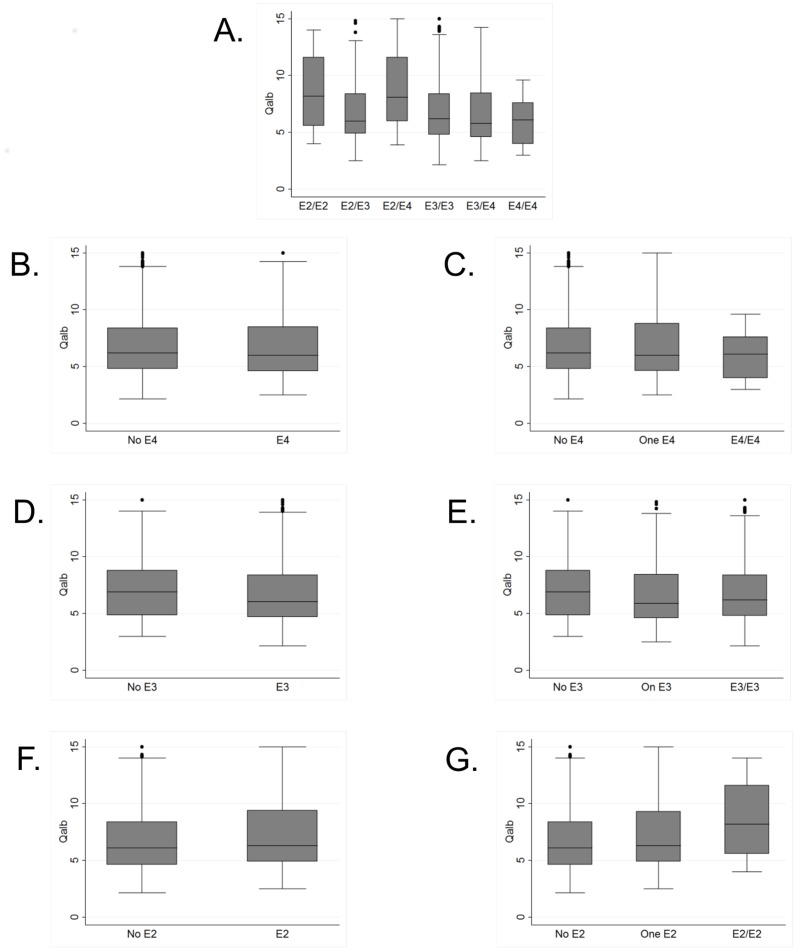
Q_Alb_ values and ApoE genotypes in a population of patients with different types of dementia (n = 520). Displayed are boxplots (box: interquartile range, line: median) of Q_Alb_ (non-logarithmised, non-transformed) values dependent on A. ApoE genotypes, B. presence of ApoE E4 alleles (dominant genetic model with E4 as risk allele), C. number of ApoE E4 alleles (0, 1 or 2, additive genetic model with E4 as risk allele), D. presence of ApoE E3 alleles (dominant genetic model with E3 as risk allele), E. number of ApoE E3 alleles (0, 1 or 2, additive genetic model with E3 as risk allele), F. presence of ApoE E2 alleles (dominant genetic model with E2 as risk allele), G. number of ApoE E2 alleles (0, 1 or 2, additive genetic model with E2 as risk allele). Corresponding summary statistics of logarithmised Q_Alb_ values can be found in Table 2 (A.) and 3 (B., C., D., E., F., G.).

In the primary analysis of this study, only weak evidence for global differences of Q_Alb_ between ApoE genotypes could be shown (p = 0.104, adjusted for age and sex, Figure 1, Table 2). Q_Alb_ values were highest in genotypes without E3 allele and lowest in E4/E4 patients. Subgroup analysis restricted to CJD patients showed the same trend (p = 0.052), whereas there was no evidence for differences between ApoE genotypes in AD (p = 0.335) and VD patients (p = 0.638, Table 2). Tukey-Post-hoc tests for pairwise comparisons did neither show significant differences between any two genotypes in the entire study population nor in the three disease subgroups.

**Table 2:** Q_Alb_ * by ApoE genotype and type of dementia.

		ApoE genotype					
		E2/E2 (n = 5)	E2/E3 (n = 59)	E2/E4 (n = 13)	E3/E3 (n = 284)	E3/E4 (n = 133)	E4/E4 (n = 26)
All cases	Mean (95% CI)	7.85 (5.00–12.34)	6.17 (5.50–6.92)	8.12 (6.43–10.24)	6.17 (5.89–6.51)	6.12 (5.70–6.57)	5.70 (4.94–6.58)
	p value**	0.104					
		E2/E2 (n = 4)	E2/E3 (n = 41)	E2/E4 (n = 8)	E3/E3 (n = 214)	E3/E4 (n = 72)	E4/E4 (n = 8)
CJD (n = 350)	Mean (95% CI)	9.29 (6.25–13.82)	6.21 (5.38–7.16)	7.94 (5.84–10.77)	6.06 (5.72–6.43)	5.56 (5.08–6.10)	5.72 (4.26–7.68)
	p value**	0.052					
		E2/E2 (n = 0)	E2/E3 (n = 5)	E2/E4(n = 4)	E3/E3 (n = 23)	E3/E4 (n = 32)	E4/E4 (n = 10)
AD (n = 71)	Mean (95% CI)	-	4.60 (3.48–6.10)	9.59 (6.43–14.32)	5.87 (4.91–7.03)	6.71 (5.75–7.82)	5.38 (4.17–6.95)
	p value**	0.335					
		E2/E2 (n = 1)	E2/E3 (n = 13)	E2/E4(n = 1)	E3/E3 (n = 47)	E3/E4 (n = 29)	E4/E4 (n = 8)
VD (n = 99)	Mean (95% CI)	4.00	6.32 (5.22–7.66)	5.00	6.97 (6.25–7.78)	7.01 (6.09–8.07)	6.11 (4.90–7.60)
	p value**	0.638					

*Back-transformed means and 95% confidence intervals of the natural logarithm of Q_Alb_.

**ANCOVA adjusted for age and sex.

In the secondary analysis of this study, the effect of ApoE alleles on Q_Alb_ was investigated using additive and dominant genetic models. Independently of the model assumed we did not find an association between the presence of one of the three ApoE alleles and Q_Alb_ in the entire study population (Table 3). However, when restricting analysis to the subgroup of CJD patients, patients without E3 allele showed higher Q_Alb_ values than those with E3 allele when using a dominant (p = 0.035) or an additive genetic model (p = 0.086). These results provide some evidence for a protective effect of the E3 allele in this patient group. This finding could not be confirmed in the subgroups of AD and VD patients (Table 3). When using either E2 or E4 as a risk allele for dominant or additive models, there was no relevant difference in Q_Alb_ between the respective genetic groups. However, Q_Alb_ values of patients with E4/E4 genotype were lower than those of patients with one or no E4 allele in all subgroups. Intra-group heterogeneity was high in all analyses, so that the variability in Q_Alb_ attributable to ApoE genotype was consistently low (R^2^ between 0 and 0.02).

**Table 3:** Q_Alb_ * by number of ApoE4, ApoE3, ApoE2 alleles and type of dementia under the assumption of dominant and additive genetic models.

	Dominant genetic model				Additive genetic model			
	Mean Q_Alb_ (95% Confidence Intervall)*				Mean Q_Alb_ (95% Confidence Intervall)*			
***E4 vs. no E4***	**≥one E4 allele**	**No E4 allele**	**P value** **	***E4 as risk allele***	**E4/E4**	**One E4 allele**	**No E4**	**P value** **
*All cases (n = 520)*	6.18 (5.81–6.58)	6.21 (5.93–6.50)	0.920	*All cases (n = 520)*	5.70 (4.94–6.58)	6.28 (5.86–6.72)	6.21 (5.93–6.50)	0.599
*CJD (n = 350)*	5.76 (5.28–6.28)	6.13 (5.81–6.47)	0.427	*CJD (n = 350)*	5.72 (4.26–7.68)	5.76 (5.27–6.31)	6.13 (5.81–6.47)	0.723
*AD (n = 71)*	6.60 (5.79–7.51)	5.76 (4.87–6.81)	0.254	*AD (n = 71)*	5.38 (4.17–6.95)	6.98 (6.03–8.08)	5.76 (4.87–6.81)	0.222
*VD (n = 99)*	6.75 (6.00–7.59)	6.77 (6.16–7.44)	0.926	*VD (n = 99)*	6.11 (4.90–7.60)	6.93 (6.04–7.95)	6.77 (6.16–7.44)	0.813
***E3 vs. no E3***	**≥one E3 allele**	**No E3 allele**	**P value** **	***E3 as risk allele***	**E3/E3**	**One E3 allele**	**No E3**	**P value** **
*All cases (n = 520)*	6.17 (5.93–6.41)	6.56 (5.78–7.46)	0.234	*All cases (n = 520)*	6.19 (5.89–6.51)	6.13 (5.78–6.51)	6.56 (5.78–7.46)	0.813
*CJD (n = 350)*	5.97 (5.70–6.26)	7.18 (5.89–8.76)	0.035	*CJD (n = 350)*	6.06 (5.72–6.43)	5.80 (5.36–6.27)	7.18 (5.89–8.76)	0.086
*AD (n = 71)*	6.27 (5.60–7.03)	6.35 (4.93–8.17)	0.921	*AD (n = 71)*	5.87 (4.91–7.03)	6.56 (5.66–7.61)	6.35 (4.93–8.17)	0.567
*VD (n = 99)*	6.89 (6.37–7.45)	5.74 (4.72–6.97)	0.274	*VD (n = 99)*	6.97 (6.25–7.78)	6.79 (6.06–7.61)	5.74 (4.72–6.97)	0.490
***E2 vs. no E2***	**≥one E2 allele**	**No E2 allele**	**P value** **	***E2 as risk allele***	**E2/E2**	**E2**	**No E2**	**P value** **
*All cases (n = 520)*	6.56 (5.93–7.27)	6.14 (5.90–6.38)	0.202	*All cases (n = 520)*	7.85 (5.00–12.34)	6.48 (5.84–7.20)	6.14 (5.90–6.38)	0.198
*CJD (n = 350)*	6.62 (5.83–7.50)	5.93 (5.65–6.22)	0.126	*CJD (n = 350)*	9.29 (6.25–13.82)	6.45 (5.66–7.34)	5.93 (5.65–6.22)	0.095
*AD (n = 71)*	7.51 (5.00–11.27)	6.19 (5.56–6.88)	0.274	*AD (n = 71)*	-	7.51 (5.00–11.27)	6.19 (5.56–6.88)	0.277
*VD (n = 99)*	6.04 (5.05–7.21)	6.90 (6.36–7.47)	0.155	*VD (n = 99)*	4.00	6.04 (5.05–7.21)	6.90 (6.36–7.47)	0.250

*Back-transformed means and 95% confidence intervals of the natural logarithm of Q_Alb_.

**ANCOVA adjusted for age and sex.

## Discussion

In the present study we investigated for the first time if ApoE genotypes are associated with BBB dysfunction estimated by cerebrospinal fluid-serum albumin ratio in a cohort of patients with different types of dementia. Within our study we did not find consistent evidence of a role of ApoE genotypes for the function of the BBB. However, there was good evidence for increased Q_Alb_ values (indicating a worse blood-brain barrier function) in CJD patients without E3 alleles potentially indicating a protective effect of E3 in this patient group. Individuals harboring an E4/E4 genotype showed generally lower Q_Alb_ values (indicating a better blood-brain barrier function). Heterogeneity of Q_Alb_ within genotypes was high, limiting the interpretability of differences observed between different genotypes.

This is the first large-sized study that investigated a potential association of ApoE with BBB dysfunction. A major strength of our study is the inclusion of different types of dementia with various progression rates. Yet, interpretation of study results is limited by low power for detecting differences between specific genotypes. Since the underlying genetic model for ApoE is unknown, we performed exploratory analyses using both dominant and additive models with different ApoE alleles as the respective risk alleles. Therefore, interpretation of the resulting p values has to take into account that multiple models have been evaluated at the same time without adjusting for it. Moreover, there was no information available about the individual course of disease or levels of cognitive dysfunction. It is not likely that this has resulted in any kind of bias, since all patients were referred to the center at time of initial diagnosis. We could not provide repeated Q_Alb_ measurements in our study since repeated lumbar punctures are rarely performed for diagnostic purposes in the study population of interest so that no information can be given about longitudinal changes in BBB function.

Another limitation is the fact that Q_Alb_ is not a perfect measurement for BBB integrity and has some limitations [22]. Most of the concerns have been raised, since Q_Alb_ increases with age in disease-free individuals [23]. Since we were not interested in actual Q_Alb_ values but in differences between groups, we accounted for this by adjusting our analyses for individual age. Investigation of BBB using molecules of different size might have offered additional information about BBB permeability. However, most of these molecules are not available in routine diagnostics. Farrall et al. concluded that using e.g. IgG index (which is available in routine diagnostics) is not suitable for measuring BBB disruption due to several reasons [24]. Gadolinium-enhanced MRI can provide a different view on BBB integrity, but was not available in a standardized way within this study. Direct measurement of BBB function e.g. via cerebral blood flow is difficult to assess in large study populations and has not been shown to provide reliable estimates of BBB function either.

Despite the fact, that albumin ratio is not a perfect marker of BBB integrity it is an easy to assess, robust and reliable standard surrogate marker that is frequently used in epidemiological studies and daily practice. Moreover, Farrall et al. pointed out in their recent systematic review that albumin ratio is still the most commonly used measure for BBB integrity and that it has been shown to “reflect BBB integrity accurately” [24]. This is supported by several timely studies covering different research topics and using albumin ratios as the method of choice for BBB integrity [25]–[27].

## Conclusion

With the present study we provide epidemiological evidence that ApoE genotypes do not play a systematic role in the development of BBB dysfunction in a large group of patients with different types of dementia. However, in the subgroup of CJD patients we did find increased Q_Alb_ values in those without E3 allele indicating a potential protective role of E3 for BBB dysfunction in this patient population. The interpretation of the results of this study must take into account that BBB function was only assessed by cerebrospinal fluid-serum albumin ratio which has been shown to be a good marker for overall BBB integrity but might not reflect all qualities of the barrier.
